# Inhibition of miR-146a Expression and Regulation of Endotoxin Tolerance by Rhesus Theta-Defensin-1

**DOI:** 10.1155/2023/8387330

**Published:** 2023-04-17

**Authors:** Prasad Tongaonkar, Katie K. Trinh, André J. Ouellette, Michael E. Selsted

**Affiliations:** ^1^Department of Pathology and Laboratory Medicine, Keck School of Medicine, University of Southern California, Los Angeles, CA, USA; ^2^USC Norris Comprehensive Cancer Center, University of Southern California, Los Angeles, CA, USA

## Abstract

Theta- (*θ-*) defensins are pleiotropic host defense peptides with antimicrobial- and immune-modulating activities. Immune stimulation of cells with lipopolysaccharide (LPS, endotoxin) activates proinflammatory gene expression and cytokine secretion, both of which are attenuated by rhesus theta-defensin-1 (RTD-1) inhibition of NF-*κ*B and MAP kinase pathways. Endotoxin tolerance is a condition that ensues when cells have an extended primary exposure to low levels of LPS, resulting in resistance to a subsequent LPS challenge. Recognition of LPS by Toll-like receptor-4 (TLR4) activates NF-*κ*B, elevating levels of microRNA-146a (miR-146a), which targets IRAK1 and TRAF6 transcripts to reduce their protein levels and inhibits TLR signaling on secondary LPS stimulation. Here, we report that RTD-1 suppressed the expression of miR-146a and stabilized the IRAK1 protein in immune-stimulated, monocytic THP-1 cells. Cells that had primary exposure to LPS became endotoxin-tolerant, as evident from their failure to secrete TNF-*α* upon secondary endotoxin challenge. However, cells incubated with RTD-1 during the primary LPS stimulation secreted TNF-*α* after secondary LPS stimulation in an RTD-1 dose-dependent manner. Consistent with this, compared to the control treatment, cells treated with RTD-1 during primary LPS stimulation had increased NF-*κ*B activity after secondary LPS stimulation. These results show that RTD-1 suppresses endotoxin tolerance by inhibiting the NF-*κ*B pathway and demonstrates a novel inflammatory role for RTD-1 that is mediated by the downregulation of miR-146a during the innate immune response.

## 1. Introduction

*θ*-defensins are a unique class of pleiotropic mammalian host defense peptides and the only known macrocyclic peptides in the animal kingdom [1]. *θ*-defensins occur exclusively in Old World monkeys, e.g., rhesus macaques [2] and baboons [3], but are absent in humans and other hominids [4]. RTD-1, the prototype *θ*-defensin, has potent microbicidal activities *in vitro* and modulates immune responses *in vitro* and *in vivo* by diverse mechanisms [5–10]. RTD-1 suppresses the secretion of proinflammatory cytokines by human blood leukocytes stimulated with diverse Toll-like receptor (TLR) ligands [7]. In immune-stimulated, differentiated THP-1 cells and human monocytes, RTD-1 reduced the expression of proinflammatory cytokine genes (*TNF*, *IL1B*, *IL8*, *CCL3*, and *CCL4*) by inhibiting the activation of NF-*κ*B and MAP kinase signaling pathways [8]. RTD-1 and related natural isoforms also reduce TNF-*α* release by inhibiting the tumor necrosis factor alpha-converting enzyme/A disintegrin and metalloproteinase 17 (TACE/ADAM17) [11] as well as matrix metalloproteinases and cathepsins involved in cellular immune responses [11, 12]. Consistent with these numerous mechanisms of action, RTD-1 promoted survival in mouse models of severe sepsis-reducing plasma levels of several cytokines and chemokines in bacteremic mice [7] and alleviated lethality in mice with systemic candidiasis [10]. Peptide treatment also moderated levels of proinflammatory cytokines in severe acute respiratory syndrome coronavirus infection [9] and in endotoxin-induced lung injury [6], and it reduced pro-inflammatory gene expression in synovial tissues in rat pristane-induced arthritis, a rodent model of rheumatoid arthritis [13].

Innate immune responses are induced when TLRs present in mammalian cells recognize pathogen-associated molecular patterns [14, 15]. LPS, present on Gram-negative bacteria, stimulates TLR4 activating the NF-*κ*B and MAP kinase pathways which initiate cellular proinflammatory responses [16]. However, prolonged exposure to low concentrations of LPS induces endotoxin tolerance in cells which confers resistance to further LPS stimulation and inhibits proinflammatory cytokine gene expression [17]. Endotoxin tolerance is regulated by multiple factors and accompanied by the reprogramming of gene expression networks [18–20]. In a previous study, a 99-gene endotoxin tolerance expression signature was identified by transcriptomic analysis of inflammatory and endotoxin-tolerant peripheral blood mononuclear cells [21]. Of note, this gene expression signature was also identified in early sepsis patients and persisted throughout the course of the disease, disclosing LPS tolerance as a factor in dysregulated immune gene expression that occurs in sepsis [21].

MicroRNAs (miRNAs) play a critical role in establishing endotoxin tolerance [22]. These small, ~23 nucleotide RNAs regulate gene expression by destabilizing mRNAs or by inhibiting their translation [23]. A single miRNA may regulate many genes and diverse cellular processes, and it is estimated that ~30% of the transcriptome may be regulated by miRNAs [24], and therefore microRNAs control diverse cellular processes [25]. Stimulation of THP-1 cells with LPS induces miR-146a by NF-*κ*B activation, and miR-146a is predicted to base pair with the 3′ untranslated regions of both IL-1R–associated kinase-1 (IRAK1) and TNF receptor-associated factor 6 (TRAF6) transcripts, thereby downregulating levels of both proteins. IRAK1 and TRAF6 are adaptor kinases downstream of TLR4 activation that promote inflammation sustained by proinflammatory cytokines. Knockdown of IRAK1 and TRAF6 in THP-1 monocytes makes them refractory to further LPS stimulation and TLR signaling [26], and these two gene products are direct miR-146a molecular targets [26].

The *in vitro* and *in vivo* immune-modulating activities of RTD-1 and its inhibition of NF-*κ*B and MAP kinase signaling pathways provided a rationale for testing whether RTD-1 regulates endotoxin tolerance. We hypothesized that RTD-1 regulates endotoxin tolerance by regulating the expression of miR-146a in LPS-stimulated cells. Accordingly, we measured the effects of RTD-1 on miR-146a expression and endotoxin tolerance markers in LPS-stimulated THP-1 cells. Our results show that RTD-1 inhibition of miR-146a expression in LPS-stimulated cells suppresses endotoxin tolerance, and this effect is mediated by RTD-1 reduction of NF-*κ*B activation.

## 2. Materials and Methods

### 2.1. Cells, Media, and Reagents

THP-1 cells obtained from the American Type Culture Collection (ATCC, Manassas, VA) were grown in RPMI-1640 medium containing 10% fetal bovine serum (FBS) and penicillin/streptomycin (p/s) [8]. THP-1 Dual cells, which express the NF-*κ*B reporter secreted embryonic alkaline phosphatase (SEAP), were obtained from InvivoGen Inc. (San Diego, CA) and grown in RPMI-1640 medium containing 10% heat-inactivated FBS and antibiotics (p/s, normocin, zeocin, and blasticidin) as recommended by InvivoGen. QUANTI-Blue (InvivoGen) and synthetic RTD-1 were dissolved in water and 0.01% acetic acid (AcOH), respectively [8]. BCA protein assay and human TNF-*α* ELISA kits were purchased from Thermo Fisher Scientific (Waltham, MA). LPS from *Salmonella enterica* serotype Minnesota was from Millipore Sigma (St. Louis, MO) and was dissolved in phosphate-buffered saline (PBS). Rabbit anti-IRAK1 monoclonal and anti-GAPDH rabbit polyclonal antibodies were from Cell Signaling Technology (Danvers, MA) and GeneTex (Irvine, CA), respectively.

### 2.2. Expression of miR-146a and Effect on IRAK1 Protein

THP-1 cells were suspended in RPMI complete medium containing 1% FBS at ~33 × 10^4^ cells/ml for 6 h and stimulated with 100 ng/ml LPS for 20 h with RTD-1 as described in the figure legends. The cells were harvested by centrifugation at 230 × g and washed with PBS. The medium was centrifuged again at 5000 × g for 5 min and analyzed using a TNF-*α* ELISA kit as per the manufacturer's instructions. Cells were used for the isolation of DNAse-treated total RNA using the Quick RNA Miniprep Kit (Zymo Research, Irvine, CA), and the RNA was further cleaned or concentrated to *A*_260/280_ > 1.8 using the RNA Clean & Concentrator-5 kit (Zymo Research). RNA was converted to first-strand cDNA using the miScript II RT kit (Qiagen, Valencia, CA). Quantitative real-time PCR (qRT-PCR) reactions were performed in duplicate using the miScript Sybr Green PCR kit (Qiagen) and miScript primer assays from Qiagen for miR-146a (Hs_miR-146a_1) and control small RNAs SNORD68 (Hs_SNORD68_11) and RNU6-2 (Hs_RNU6-2_11). The cycling parameters for qRT-PCR were 94°C for 15 s, 55°C for 15 s, and 70°C for 30 s for 40 cycles using a C1000 Thermal Cycler equipped with a CFX96 real-time system (Bio-Rad, Hercules, CA). Fold stimulation was calculated by the 2-*ΔΔ*Cq method [27], and melt curves were performed to confirm the amplification of single PCR products.

For analysis of IRAK1 protein levels, cells were lysed using cell lysis buffer (Cell Signaling Technology) containing 1 mM phenylmethylsulfonyl fluoride. Western blots were performed as described previously [8] and were scanned and quantitated using U.S. National Institutes of Health ImageJ software (Bethesda, MD, USA).

### 2.3. Effect of RTD-1 on Endotoxin Tolerance

THP-1 cells were resuspended in RPMI complete medium containing 1% FBS for 6 h as above and treated with 10 ng/ml LPS and RTD-1 for 20 h as noted in the figure legends. Cells were washed two times with PBS and stimulated with PBS (control) or 100 ng/ml LPS; 0-10 *μ*g/ml RTD-1 and TNF-*α* released into the medium were analyzed by ELISA.

THP-1 Dual cells were preincubated for 2 h in RPMI complete medium containing 1% heat-inactivated (HI) FBS and then treated with PBS, 10 ng/ml LPS, or 10 *μ*g/ml RTD-1 + 10 ng/ml LPS for 18 h. The cells were then washed twice with complete medium containing 1% HI-FBS, and cells were stimulated with either PBS or 100 ng/ml LPS for 22 h. The medium was then analyzed for NF-*κ*B activation by measuring SEAP activity using the QUANTI-Blue assay.

### 2.4. Statistical Analysis

Paired *t* tests of mean ± standard deviation from 3 experiments were plotted and analyzed using Excel. *P* < 0.05 was considered significant.

## 3. Results

### 3.1. RTD-1 Inhibits Expression of miR-146a in LPS-Stimulated THP-1 Cells

**Figure 1:**
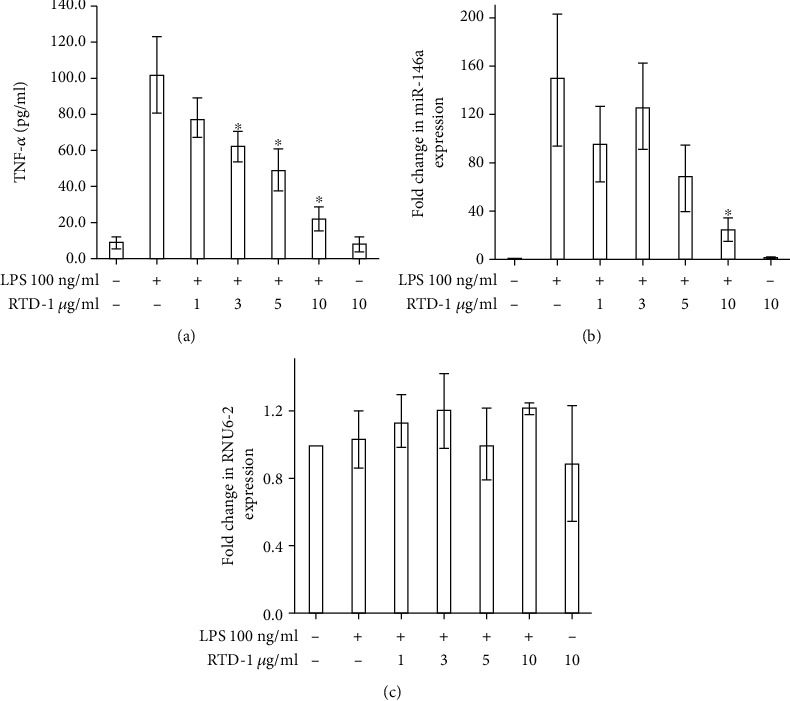
RTD-1 inhibits expression of miR-146a in LPS-stimulated cells. THP-1 cells were treated with 100 ng/ml LPS and RTD-1 at the indicated concentrations for 20 h. The medium was analyzed by TNF-*α* ELISA (a), and the RNA was analyzed by qRT-PCR for expression of miR-146a (b), and RNU6-2 (c) after normalizing to SNORD68 expression. Data shown are the results of 3 experiments; ^∗^*P* < 0.05 compared to LPS treatment, two-tailed, paired *t* test. Fold change was calculated with respect to vehicle-treated control in (b) and (c).

**Figure 2:**
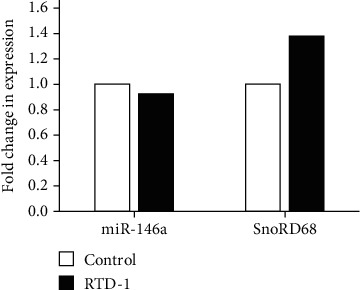
RTD-1 does not affect miR-146a stability. THP-1 cells were incubated with 10 ng/ml LPS for 18 h to induce the expression of miR-146a. The cells were then washed and treated with vehicle or 10 *μ*g/ml RTD-1 for 4 h. The RNA was then analyzed by qRT-PCR for miR-146a and SnoRD68 expression after normalizing it to RNU6-2 expression. The average of the 2 experiments is shown. Fold change was calculated with respect to control.

RTD-1 concentration-dependently inhibited TNF-*α* secretion by THP-1 cells stimulated with *Salmonella enterica* LPS (Figure 1(a)). LPS stimulation increased the expression of miR-146a, and expression of this miRNA was inhibited by RTD-1 in a concentration-dependent manner (Figure 1(b)). In contrast, levels of control small RNA RNU6-2 were not affected by RTD-1 treatment of LPS-stimulated cells (Figure 1(c)). To test whether the regulation of miR-146a levels by RTD-1 was posttranscriptional, THP-1 cells were incubated with LPS for 18 h to induce miR-146a and only then treated with RTD-1. Under these conditions, RTD-1 did not decrease cellular miR-146a levels compared to untreated controls, showing that RTD-1 regulated miR-146a by reducing gene expression in LPS-stimulated cells as opposed to inducing transcript instability (Figure 2).

### 3.2. Stabilization of IRAK1 by RTD-1 in LPS-Treated Cells

**Figure 3:**
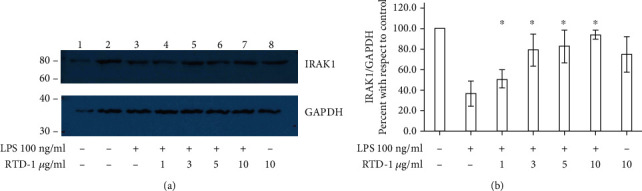
RTD-1 stabilizes IRAK1 in LPS-stimulated cells. THP-1 cells were treated with 100 ng/ml LPS in the presence of RTD-1 for 20 h as shown. (a) Extracts from cells were analyzed by western blotting experiments using the anti-IRAK1 and anti-GAPDH antibodies. A representative western blot from three experiments is shown. The sample in lane 1 is the same as that in control lane 2 but has 50% less protein. The numerals at the left indicate molecular weights in kDa. (b) Quantification of western blots from 3 experiments. ^∗^*P* < 0.05 compared to LPS treatment, two-tailed, paired *t* test.

miR-146a induction contributes to the establishment of endotoxin tolerance by targeting IRAK1, leading to inhibition of TLR signaling and rendering cells refractory to subsequent LPS challenge [28]. Agonist stimulation of TLR pathways activates IRAK1 kinase with subsequent NF-*κ*B pathway activation [15] which induces miR-146a expression which in turn reduces levels of IRAK1 protein [26, 29]. As shown in Figures 3(a) and 3(b), levels of the IRAK1 protein declined by more than 60% in cells exposed to LPS for 20 h. However, RTD-1 treatment upregulated the IRAK1 protein in a concentration-dependent fashion, with 10 *μ*g/ml RTD-1 restoring IRAK1 to control levels. IRAK1 levels were not significantly altered by RTD-1 in cells that were not stimulated with LPS (Figure 3).

### 3.3. RTD-1 Suppresses Endotoxin Tolerance

**Figure 4:**
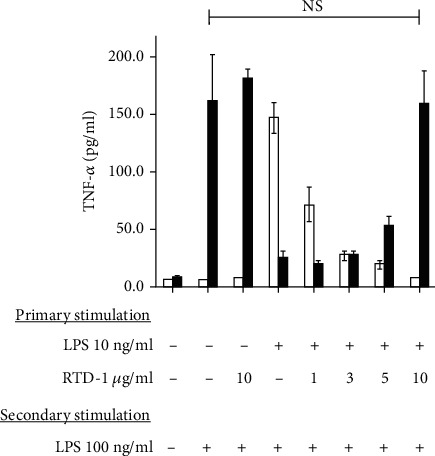
RTD-1 inhibits the establishment of endotoxin tolerance. THP-1 cells were incubated with 10 ng/ml LPS and RTD-1 for 20 h as indicated. Cells were washed and restimulated with 100 ng/ml LPS for 4 h. TNF-*α* secretion in the medium was analyzed by ELISA after primary stimulation (white columns) and secondary stimulation (black columns). The average of 3 experiments is shown; NS: nonsignificant, *P* = 0.949 two-tailed, paired *t* test. Primary LPS exposure suppresses endotoxin tolerance.

TNF-*α* secretion is suppressed in endotoxin tolerance [26, 28]. We therefore analyzed the effect of RTD-1 on the secretion of TNF-*α* by LPS-treated THP-1 cells. THP-1 cells were treated for 20 h with a primary low dose (10 ng/ml) of LPS in combination with varied levels of RTD-1. As before (Figure 1), RTD-1 treatment suppressed TNF-*α* release during primary LPS exposure (Figure 4). Following primary stimulation, cells were washed and once again stimulated with LPS for 4 h, and secreted TNF-*α* was quantified (Figure 4). Control cells that had not been exposed to primary low-dose LPS responded to secondary LPS stimulation by robust secretion of TNF-*α*. As expected, THP-1 cells first stimulated with 10 ng/ml LPS secreted markedly lower amounts of TNF-*α* upon secondary LPS stimulation, the hallmark of endotoxin tolerance. In contrast, cells initially coincubated with LPS and RTD-1, then washed and once again stimulated with LPS, secreted increased levels of TNF-*α* as a function of RTD-1 concentration utilized in the primary incubation (Figure 4). Regardless of the timing of endotoxin stimulation, cells treated with LPS alone secreted high levels of TNF-*α*. Thus, coincubation of RTD-1 and LPS prevented THP-1 cells from becoming refractory to endotoxin (Figure 4).

### 3.4. RTD-1 Downregulation of the NF-*κ*B Pathway Inhibits Endotoxin Tolerance

**Figure 5:**
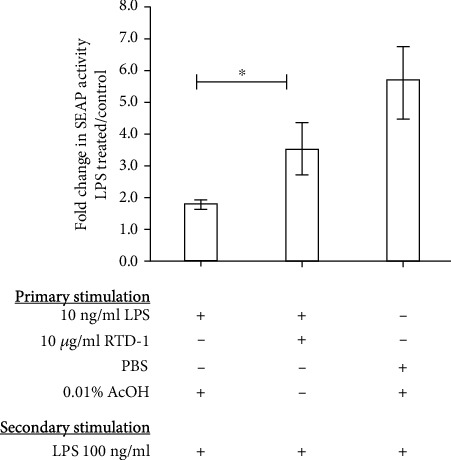
RTD-1 inhibits endotoxin tolerance by regulating NF-*κ*B activity. THP-1 Dual cells were treated with 10 ng/ml LPS, LPS+10 *μ*g/ml RTD-1, or PBS for 18 h. The cells were washed and restimulated with vehicle control or 100 ng/ml LPS for ~22 h. Supernatant SEAP activity was measured by the QUANTI-Blue assay and the fold change between LPS and vehicle-treated secondary stimulation from 3 experiments was plotted. ^∗^*P* < 0.05 one-tailed, paired *t* test.

As noted above, RTD-1 moderates the proinflammatory responses of THP-1 cells stimulated by diverse TLR ligands, in part by inhibiting the NF-*κ*B pathway [8]. Therefore, we analyzed the effect of RTD-1 on NF-*κ*B activation in THP-1 Dual cells exposed to LPS once (10 ng/ml for 18 h) or twice (10 ng/ml for 18 h plus 100 ng/ml for 22 h). NF-*κ*B pathway activity was measured by quantifying SEAP activity in the medium (Methods). Stimulation of cells with 100 ng/ml LPS alone activated NF-*κ*B~six fold (Figure 5). NF-*κ*B activity was reduced by 70% in cells that were first stimulated with 10 ng/ml LPS and subsequently treated with 100 ng/ml LPS. In contrast, coincubation with RTD-1 during primary LPS stimulation resulted in greater NF-*κ*B activity than cells stimulated with LPS in the absence of RTD-1 (Figure 5; *P* < 0.05). Thus, RTD-1 inhibition of NF-*κ*B activation during primary LPS exposure suppresses endotoxin tolerance.

## 4. Discussion

**Figure 6:**
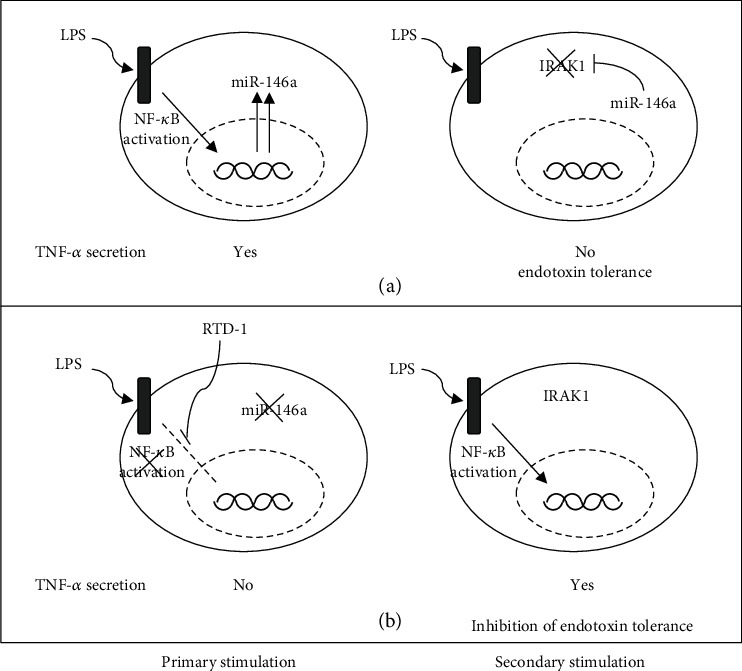
Effect of RTD-1 on miR-146a expression and endotoxin tolerance. (a) LPS stimulation of cells leads to activation of the NF-*κ*B pathway and miR-146a expression. IRAK1 is targeted by miR-146a which inhibits further stimulation of the TLR pathway. (b) RTD-1 inhibits activation of the NF-*κ*B pathway which leads to inhibition of miR-146a expression. Suppression of miR-146a expression stabilizes IRAK1 and enables a cellular proinflammatory response as observed by TNF-*α* secretion during secondary stimulation with LPS.

We show that RTD-1 regulates the expression of the microRNA, miR-146a. RTD-1 attenuation of NF-*κ*B activation during primary LPS stimulation of THP-1 monocytic cells leads to reduced miR-146a levels, which enable secondary LPS stimulation and the secretion of TNF-*α* (Figure 6). Previous studies indicate that miR-146a regulates IRAK1 protein expression [26]. However, changes in the expression of IRAK1 protein do not correlate with steady-state IRAK1 mRNA levels [28, 30, 31]. In the THP-1 cells subjected to LPS primary stimulation, RTD-1 inhibited miR-146a expression and stabilized cellular IRAK1 protein levels. LPS-stimulated THP-1 cells incubated with RTD-1 retained the ability to respond to secondary LPS stimulation as evidenced by TNF secretion in these cells, consistent with its ability to block resistance to endotoxin.

Here, we have investigated one aspect of endotoxin tolerance; however, the establishment and maintenance of endotoxin tolerance are regulated at multiple levels. These include the formation of lipid rafts for signaling through the TLR4 receptor [32], regulation of phosphorylation of signaling transduction mediators [33–35], regulation of gene expression by the formation of repressive p50 homodimers [36, 37], chromatin remodeling [38, 39], and demethylation [35, 40, 41]. The NF-*κ*B family gene, RELB, is upregulated upon LPS stimulation and is involved in the transcription of I*κ*B*α*, a repressor of NF-*κ*B, and inhibits expression of the TNF and IL1B genes [42, 43]. In our studies, RELB expression was upregulated upon stimulation of THP-1 cells with LPS, but this LPS-dependent stimulation of RELB was unaffected by the addition of RTD-1 (data not shown). This suggests that the RTD-1 effect on endotoxin tolerance is not mediated through the regulation of RELB mRNA levels and is not simply a result of the sequestration of LPS by RTD-1. RTD-1 regulates gene expression and immune signaling pathways in myeloid cells [8] and in synovial tissues of a rat model of rheumatoid arthritis [14], and it reduced lethality in mouse models of polymicrobial [7] and *Candida albicans* [10] sepsis. The cellular theta-defensin receptor/s that mediates these diverse effects is not known and is an active area of research.

*In vivo*, endotoxin tolerance renders the host susceptible to overwhelming secondary infections in unresolved sepsis. RTD-1 suppresses acute inflammation by inhibiting TNF-*α* secretion from cells stimulated with diverse TLR agonists [7, 8], and as shown here, it promotes protective immune activation in an *in vitro* model of endotoxin tolerance. We speculate that, *in vivo*, the effects of RTD-1 may be modulated by the immune activation status of the cell, and that, by regulating miR-146a expression, RTD-1 may fine-tune the cellular immune response. To our knowledge, regulation of miRNA expression by a primate defensin is novel and provides new mechanistic insights into the pleiotropic functions of RTD-1.

## 5. Conclusion

Inhibition of miR-146a expression by RTD-1 during primary endotoxin stimulation inhibits the establishment of endotoxin tolerance in THP-1 cells and allows a cellular proinflammatory response during secondary LPS stimulation.

## Data Availability

Data is available on request.

## References

[1] Selsted M. E., Ouellette A. J. (2005). Mammalian defensins in the antimicrobial immune response. *Nature Immunology*.

[2] Tang Y. Q., Yuan J., Osapay G. (1999). A cyclic antimicrobial peptide produced in primate leukocytes by the ligation of two truncated alpha-defensins. *Science*.

[3] Garcia A. E., Osapay G., Tran P. A., Yuan J., Selsted M. E. (2008). Isolation, synthesis, and antimicrobial activities of naturally occurring theta-defensin isoforms from baboon leukocytes. *Infection and Immunity*.

[4] Nguyen T. X., Cole A. M., Lehrer R. I. (2003). Evolution of primate *θ*-defensins: a serpentine path to a sweet tooth. *Peptides*.

[5] Bensman T. J., Jayne J. G., Sun M. (2017). Efficacy of rhesus theta-defensin-1 in experimental models of Pseudomonas aeruginosa lung infection and inflammation. *Antimicrobial Agents and Chemotherapy*.

[6] Jayne J. G., Bensman T. J., Schaal J. B. (2018). Rhesus *θ*-defensin-1 attenuates endotoxin-induced acute lung injury by inhibiting proinflammatory cytokines and neutrophil recruitment. *American Journal of Respiratory Cell and Molecular Biology*.

[7] Schaal J. B., Tran D., Tran P. (2012). Rhesus macaque theta defensins suppress inflammatory cytokines and enhance survival in mouse models of bacteremic sepsis. *PLoS One*.

[8] Tongaonkar P., Trinh K. K., Schaal J. B. (2015). Rhesus macaque *θ*-defensin RTD-1 inhibits proinflammatory cytokine secretion and gene expression by inhibiting the activation of NF-*κ*B and MAPK pathways. *Journal of Leukocyte Biology*.

[9] Wohlford-Lenane C. L., Meyerholz D. K., Perlman S. (2009). Rhesus theta-defensin prevents death in a mouse model of severe acute respiratory syndrome coronavirus pulmonary disease. *Journal of Virology*.

[10] Basso V., Tran D. Q., Schaal J. B. (2019). Rhesus theta defensin 1 promotes long term survival in systemic candidiasis by host directed mechanisms. *Scientific Reports*.

[11] Schaal J. B., Maretzky T., Tran D. Q. (2018). Macrocyclic *θ*-defensins suppress tumor necrosis factor-*α* (TNF-*α*) shedding by inhibition of TNF-*α*-converting enzyme. *The Journal of Biological Chemistry*.

[12] Schaal J. B., Tran D. Q., Subramanian A. (2017). Suppression and resolution of autoimmune arthritis by rhesus *θ*-defensin-1, an immunomodulatory macrocyclic peptide. *PLoS One*.

[13] Tongaonkar P., Punj V., Subramanian A. (2019). RTD-1 therapeutically normalizes synovial gene signatures in rat autoimmune arthritis and suppresses proinflammatory mediators in RA synovial fibroblasts. *Physiological Genomics*.

[14] Akira S., Hemmi H. (2003). Recognition of pathogen-associated molecular patterns by TLR family. *Immunology Letters*.

[15] Akira S. (2009). Pathogen recognition by innate immunity and its signaling. *Proceedings of the Japan Academy. Series B, Physical and Biological Sciences*.

[16] Akira S. (2006). TLR signaling. *Current Topics in Microbiology and Immunology*.

[17] West M. A., Heagy W. (2002). Endotoxin tolerance: a review. *Critical Care Medicine*.

[18] Biswas S. K., Lopez-Collazo E. (2009). Endotoxin tolerance: new mechanisms, molecules and clinical significance. *Trends in Immunology*.

[19] Cavaillon J. M., Adib-Conquy M. (2006). Bench-to-bedside review: endotoxin tolerance as a model of leukocyte reprogramming in sepsis. *Critical Care*.

[20] Shalova I. N., Lim J. Y., Chittezhath M. (2015). Human monocytes undergo functional re-programming during sepsis mediated by hypoxia-inducible factor-1*α*. *Immunity*.

[21] Pena O. M., Hancock D. G., Lyle N. H. (2014). An endotoxin tolerance signature predicts sepsis and organ dysfunction at initial clinical presentation. *eBioMedicine*.

[22] Nahid M. A., Satoh M., Chan E. K. (2011). MicroRNA in TLR signaling and endotoxin tolerance. *Cellular & Molecular Immunology*.

[23] Bartel D. P. (2009). MicroRNAs: target recognition and regulatory functions. *Cell*.

[24] Taganov K. D., Boldin M. P., Baltimore D. (2007). MicroRNAs and immunity: tiny players in a big field. *Immunity*.

[25] Inui M., Martello G., Piccolo S. (2010). MicroRNA control of signal transduction. *Nature Reviews. Molecular Cell Biology*.

[26] Taganov K. D., Boldin M. P., Chang K. J., Baltimore D. (2006). NF-kappaB-dependent induction of microRNA miR-146, an inhibitor targeted to signaling proteins of innate immune responses. *Proceedings of the National Academy of Sciences of the United States of America*.

[27] Livak K. J., Schmittgen T. D. (2001). Analysis of relative gene expression data using real-time quantitative PCR and the 2^−*ΔΔC*^_T_ method. *Methods*.

[28] Nahid M. A., Pauley K. M., Satoh M., Chan E. K. (2009). Implication in innate immunity. *The Journal of Biological Chemistry*.

[29] Nahid M. A., Satoh M., Chan E. K. (2011). Mechanistic role of microRNA-146a in endotoxin-induced differential cross-regulation of TLR signaling. *Journal of Immunology*.

[30] Adib-Conquy M., Cavaillon J. M. (2002). Gamma interferon and granulocyte/monocyte colony-stimulating factor prevent endotoxin tolerance in human monocytes by promoting interleukin-1 receptor- associated kinase expression and its association to MyD88 and not by modulating TLR4 expression. *The Journal of Biological Chemistry*.

[31] Siedlar M., Frankenberger M., Benkhart E. (2004). Tolerance induced by the lipopeptide Pam3Cys is due to ablation of IL-1R-associated kinase-1. *Journal of Immunology*.

[32] Cuschieri J., Billigren J., Maier R. V. (2006). Endotoxin tolerance attenuates LPS-induced TLR4 mobilization to lipid rafts: a condition reversed by PKC activation. *Journal of Leukocyte Biology*.

[33] Xiong Y., Murphy M., Manavalan T. T. (2016). Endotoxin tolerance inhibits Lyn and c-Src phosphorylation and association with toll-like receptor 4 but increases expression and activity of protein phosphatases. *Journal of Innate Immunity*.

[34] Medvedev A. E., Kopydlowski K. M., Vogel S. N. (2000). Inhibition of lipopolysaccharide-induced signal transduction in endotoxin-tolerized mouse macrophages: dysregulation of cytokine, chemokine, and toll-like receptor 2 and 4 gene expression. *Journal of Immunology*.

[35] Morante-Palacios O., Lorente-Sorolla C., Ciudad L. (2021). JAK2-STAT epigenetically regulates tolerized genes in monocytes in the first encounter with gram-negative bacterial endotoxins in sepsis. *Frontiers in Immunology*.

[36] Kastenbauer S., Ziegler-Heitbrock H. W. (1999). NF-kappaB1 (p50) is upregulated in lipopolysaccharide tolerance and can block tumor necrosis factor gene expression. *Infection and Immunity*.

[37] Ziegler-Heitbrock L. (2001). The p50-homodimer mechanism in tolerance to LPS. *Journal of Endotoxin Research*.

[38] Chen J., Ivashkiv L. B. (2010). IFN-*γ* abrogates endotoxin tolerance by facilitating toll-like receptor-induced chromatin remodeling. *Proceedings of the National Academy of Sciences of the United States of America*.

[39] Foster S. L., Hargreaves D. C., Medzhitov R. (2007). Gene-specific control of inflammation by TLR-induced chromatin modifications. *Nature*.

[40] Zhang Q., Cao X. (2021). Epigenetic remodeling in innate immunity and inflammation. *Annual Review of Immunology*.

[41] Vachharajani V., McCall C. E. (2019). Epigenetic and metabolic programming of innate immunity in sepsis. *Innate Immunity*.

[42] Chen X., Yoza B. K., El Gazzar M., Hu J. Y., Cousart S. L., McCall C. E. (2009). RelB sustains IkappaBalpha expression during endotoxin tolerance. *Clinical and Vaccine Immunology*.

[43] Yoza B. K., Hu J. Y., Cousart S. L., Forrest L. M., McCall C. E. (2006). Induction of RelB participates in endotoxin tolerance. *Journal of Immunology*.

